# Bootstrapping BI-RADS classification using large language models and transformers in breast magnetic resonance imaging reports

**DOI:** 10.1186/s42492-025-00189-8

**Published:** 2025-04-03

**Authors:** Yuxin Liu, Xiang Zhang, Weiwei Cao, Wenju Cui, Tao Tan, Yuqin Peng, Jiayi Huang, Zhen Lei, Jun Shen, Jian Zheng

**Affiliations:** 1 School of Biomedical Engineering (Suzhou), University of Science and Technology of China, Division of Life Sciences and Medicine, Hefei, 230026 Anhui China; 2 Medical Imaging Department, Suzhou Institute of Biomedical Engineering and Technology, Chinese Academy of Sciences, Suzhou, 215163 Jiangsu China; 3 Department of Radiology, Sun Yat-Sen Memorial Hospital, Sun Yat-Sen University, Guangzhou, 510120 Guangdong China; 4 Guangdong Provincial Key Laboratory of Malignant Tumor Epigenetics and Gene Regulation, Medical Research Center, Sun Yat-Sen Memorial Hospital, Sun Yat-Sen University, Guangzhou, 510120 Guangdong China; 5 Shandong Laboratory of Advanced Biomaterials and Medical Devices in Weihai, Shandong University, Weihai, 264200 Shandong China; 6 Faculty of Applied Sciences, Macao Polytechnic University, Macao, China; 7 Institute of Automation, Chinese Academy of Sciences, Beijing, 100190 China

**Keywords:** Large language model, Structured report, Missing category information, Radiology report

## Abstract

Breast cancer is one of the most common malignancies among women globally. Magnetic resonance imaging (MRI), as the final non-invasive diagnostic tool before biopsy, provides detailed free-text reports that support clinical decision-making. Therefore, the effective utilization of the information in MRI reports to make reliable decisions is crucial for patient care. This study proposes a novel method for BI-RADS classification using breast MRI reports. Large language models are employed to transform free-text reports into structured reports. Specifically, missing category information (MCI) that is absent in the free-text reports is supplemented by assigning default values to the missing categories in the structured reports. To ensure data privacy, a locally deployed Qwen-Chat model is employed. Furthermore, to enhance the domain-specific adaptability, a knowledge-driven prompt is designed. The Qwen-7B-Chat model is fine-tuned specifically for structuring breast MRI reports. To prevent information loss and enable comprehensive learning of all report details, a fusion strategy is introduced, combining free-text and structured reports to train the classification model. Experimental results show that the proposed BI-RADS classification method outperforms existing report classification methods across multiple evaluation metrics. Furthermore, an external test set from a different hospital is used to validate the robustness of the proposed approach. The proposed structured method surpasses GPT-4o in terms of performance. Ablation experiments confirm that the knowledge-driven prompt, MCI, and the fusion strategy are crucial to the model’s performance.

**Supplementary Information:**

The online version contains supplementary material available at 10.1186/s42492-025-00189-8.

## Introduction

Breast cancer is one of the most prevalent malignant tumors in women worldwide and imposes a significant health burden [1]. In the diagnostic pathway, magnetic resonance imaging (MRI) represents the final non-invasive diagnostic method before considering a biopsy, which may present risks such as bleeding and complications [2, 3]. Computer-aided decision support assists less-experienced specialists while reducing unnecessary biopsies and minimizing the pathologists’ workload [4–7]. Considering their comprehensive medical information content, breast MRI reports play a crucial role in clinical decision-making. Consequently, developing effective methods to extract and learn key features from these reports shows significant potential to improve the accuracy of decision-making in breast BI-RADS classification, particularly in differentiating between malignant (suggestion for biopsy) and benign (suggestion for follow-up).

Advancement of radiology report classification through natural language processing (NLP) approaches has become increasingly important [8, 9]. Traditional machine learning methods [10], such as the support vector machine (SVM), k-nearest neighbor (KNN), Naive Bayes (NB), and maximum entropy classifier, although widely used in report classification, face challenges in feature extraction, particularly when dealing with the high-dimensional and sparse nature of text representations. These limitations impede the accurate capture of intricate inter-feature relationships. In contrast, deep learning methods enable direct extraction of high-level features from data. Convolutional neural network (CNN), recurrent neural network (RNN), and bidirectional long short-term memory network have achieved significant success in classifying radiology reports [11, 12]. However, these models may encounter difficulties in handling long-distance dependencies and capturing global semantic information. To address these limitations, the bidirectional encoder representations from transformers (BERT) [13] model has emerged as a breakthrough technology, demonstrating remarkable success in clinical text classification through variants such as ClinicalBERT [14], BioBERT [15], and RadBERT [16]. However, the effectiveness of these models depends heavily on high-quality [17–19] and large-scale domain-specific corpora, and limitations in data quality and evaluation methods can significantly compromise model effectiveness. Recently, large language models (LLMs) have demonstrated revolutionary potential in the medical field, particularly in diagnostic assistance, personalized treatment planning, clinical decision support, and risk prediction [20]. For medical text classification tasks, researchers have extensively explored the application of advanced models such as ChatGPT and GPT-4 in zero-, one-, and few-shot learning scenarios [21–23]. These models demonstrate rapid adaptation to new tasks with limited data, substantially reducing dependence on manual annotation. However, general-purpose LLMs face challenges because of their domain-specific accuracy. Their black-box nature makes identifying parts of the data that are crucial for classification tasks challenging, potentially limiting their reliable application in clinical settings.

Information extraction encompasses the process of identifying entities, relationships, and events in unstructured text [24]. This process organizes various data attributes, providing a foundation for recognizing and utilizing key information in radiology report classification. However, variations in radiologists’ writing styles and educational backgrounds result in inconsistencies in structured data attributes, which can cause patient confusion and impede effective physician communication [25].

To extract information from radiology reports, researchers have explored various approaches. Although rule-based NLP methods have shown effectiveness in certain scenarios, they remain language-dependent with limited generalizability [26]. The adoption of deep-learning techniques has led to significant performance improvements [27, 28]. However, these techniques require substantial amounts of manually annotated data. LLMs offer a promising solution for automatic information extraction, leveraging their advanced semantic understanding. Studies have demonstrated that the GPT-4 model successfully converts free-text reports into structured reports [29, 30]. However, the use of the GPT-4 model requires rigorous privacy measures to safeguard sensitive medical data. Furthermore, the prevalence of medical terminology in radiology reports poses significant challenges for general LLMs when performing information extraction tasks in this domain.

To address these challenges, a novel computer-aided BI-RADS classification method based on breast MRI reports is proposed, designed to assist less experienced specialists in accurately assessing the severity of breast lesions. The proposed approach converts free-text reports into structured reports and enhances their completeness by supplementing missing category information (MCI) with default values. By providing richer contextual information for model training, this approach improves the model’s ability to differentiate between the nature and severity of lesions. To ensure data privacy and strengthen the domain-specific applicability of the model, Qwen-14B-Chat was deployed locally, and a knowledge-driven prompt was developed, incorporating the fifth edition of the MRI imaging lexicon [31]. Subsequently the Qwen-7B-Chat model was fine-tuned to optimize its performance in structuring breast MRI reports. To mitigate potential information gaps during the structuring process of LLMs, a fusion strategy was designed that combines free-text and structured reports for joint training, thereby optimizing the model’s performance.

The main contributions of this study are as follows.

1. Development of privacy-preserving LLMs for Chinese breast MRI report structuring through knowledge-driven prompt and domain-specific model fine-tuning.
2. Enhancement of the learning capabilities of the model by incorporating MCI from free-text reports into structured reports.
3. Introduction of an innovative fusion strategy that synthesizes free-text and structured reports for comprehensive information processing.

## Methods

**Fig. 1:**
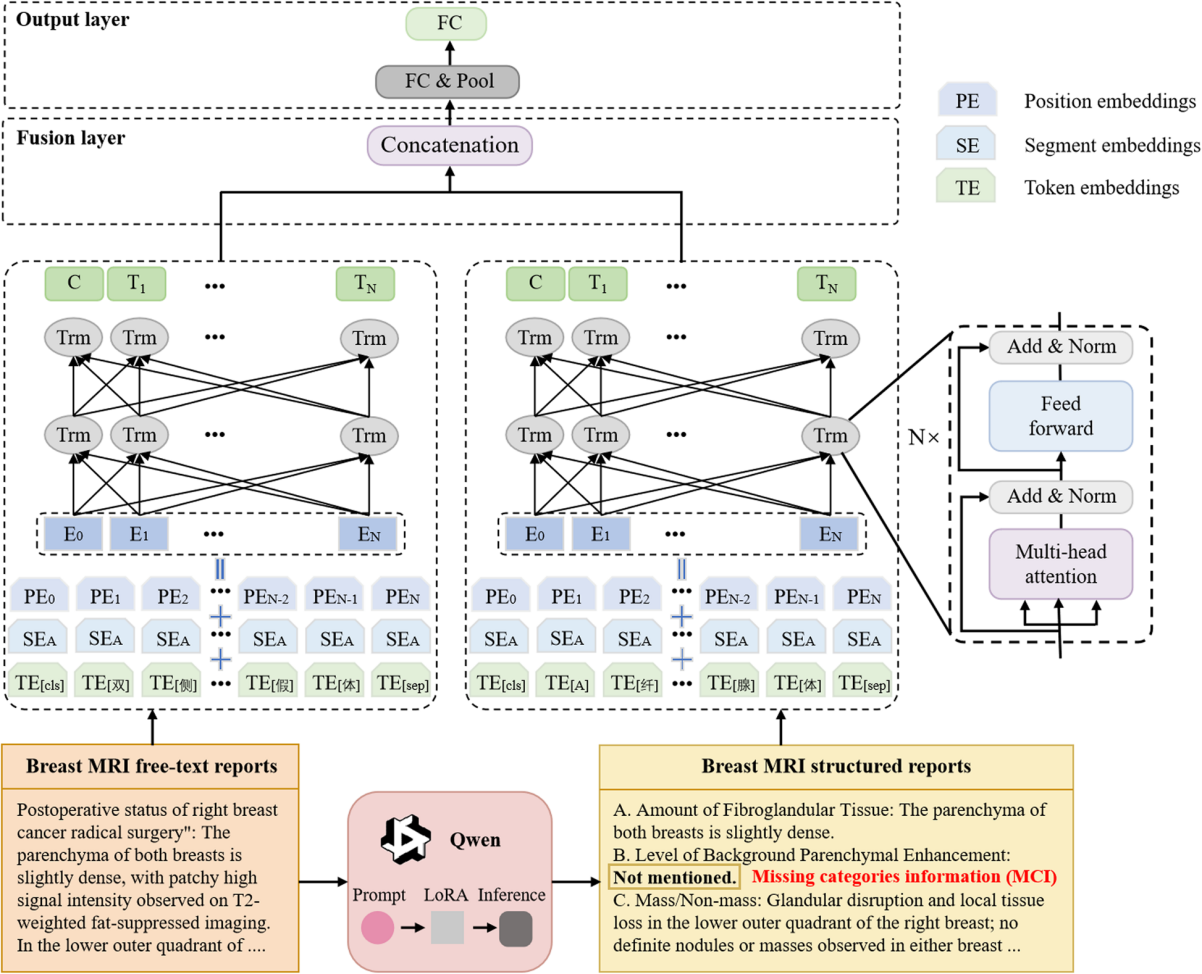
Main architecture of the proposed method. Examples of the report shown in this figure are the English translations of the original Chinese reports

This section presents a novel computer-aided BI-RADS classification method based on breast MRI reports. The methodology comprised two main stages: first, the reports were structured using LLMs, with MCI integration. Second, to mitigate potential information gaps during the structuring process, a fusion framework was developed to train the classification model, as illustrated in Fig. 1.

### Breast MRI report structuring

To ensure patient information privacy, this study utilized the locally deployed first version of the Qwen-Chat model [32], released by Alibaba in 2023 for the inference and fine-tuning experiments. This model demonstrated exceptional performance in terms of text comprehension and information extraction.

#### Knowledge-driven instruction tuning

**Fig. 2:**
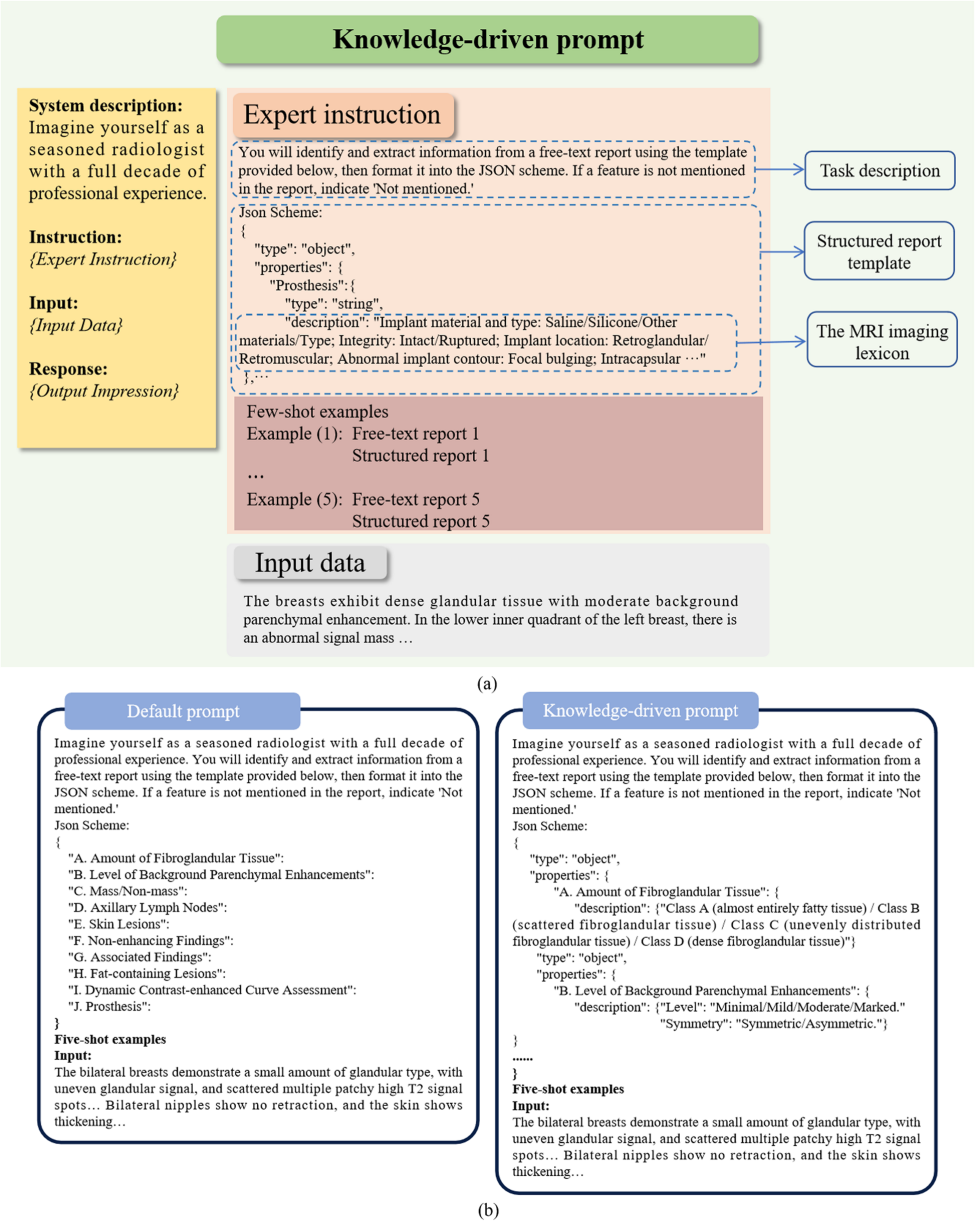
Overview of knowledge-driven prompts. **a** A knowledge-driven prompt consists of three components: system description, instruction, and input, collectively forming a complete prompt. The “Expert instruction” and “Input data” on the right side of the figure are inserted into *{Expert Instruction}* and *{Input Data}* on the left side, respectively. The generated result appears in *{Output Impression}*; **b** Illustrates the differences between knowledge-driven and default prompts for structuring breast MRI reports, where the knowledge-driven prompts provide explicit definitions for each structured category. The report examples shown in this figure are English translations of the original Chinese reports. The prompts are displayed in truncated form

According to research by Heston and Khun [33], generative language models (GLMs) possess the capability for personalized learning and timely feedback. Within the medical domain, effective utilization of GLMs requires carefully constructed task-specific prompts to generate accurate inferences. This study designed a knowledge-driven prompt that integrates the fifth edition of the MRI imaging lexicon [31] to enhance the model’s comprehension, learning, and reasoning abilities. Figure 2a illustrates the knowledge-driven prompt designed in this study, which consists of three main parts: system description, instruction, and input. The system description defines the model’s identity and behavior. The instruction provides guidance for structured information extraction, including a task description, a structured report template with the MRI imaging lexicon, and five example reports with expected responses. The input section contains the “radiological description” content of the breast MRI reports. The response section consists of structured reports generated by the model. Figure 2b highlights the key distinction between knowledge-driven and default prompts, which lies in the incorporation of the MRI imaging lexicon within the structured report template.

#### Low-rank adaptation

**Fig. 3:**
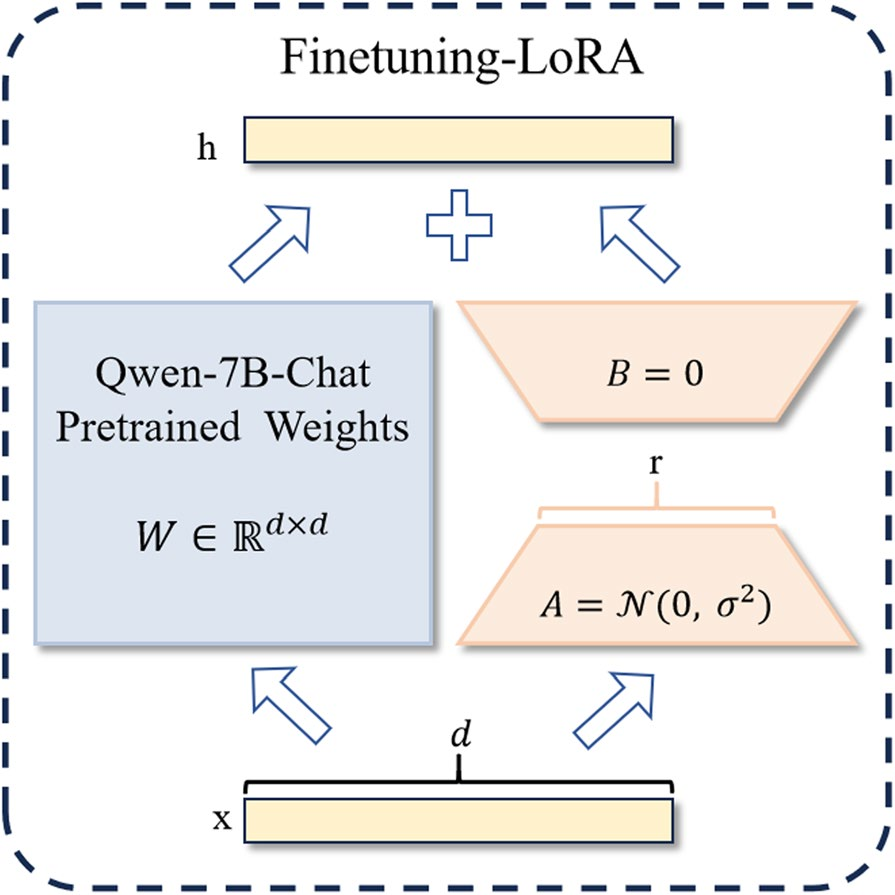
Schematic of LoRA fine-tuning

Full-parameter fine-tuning presents challenges for currently popular LLMs. Low-rank adaptation (LoRA) [34] fine-tuning method addresses modifications to the original weight matrix within the self-attention module. It employs low-rank decomposition optimization during the weight update process for downstream tasks. As illustrated in Fig. 3, during implementation, the optimized low-rank decomposition matrix is combined with the self-attention weight matrix to adjust the weights [35]. For the pre-trained weights ${W}_{0}\in \mathbb {R}^{d\times k}$ of the original language model, the weight update can be expressed as the following addition of the original weights and low-rank updates:

1 $$\begin{aligned} W_{0} + \Delta W = W_{0} + BA \end{aligned}$$

Here, *A* and *B* are the matrices of the low-rank decomposition with $B\in \mathbb {R}^{d\times r}$ and $A\in \mathbb {R}^{r\times k}$, where rank $r\ll \min \left( {d,k}\right)$. During training, ${W}_{0}$ remains frozen, whereas *A* and *B* contain trainable parameters. For ${h}= {W}_{0}{x}$, the formula for forward propagation is as follows:

2 $$\begin{aligned} h = W_{0}x + \Delta Wx = W_{0}x + BAx \end{aligned}$$

Matrix *A* is initialized with random Gaussian values, whereas *B* was initialized with zeros. At the beginning of training, the initialization of $\Delta W= BA$ is zero.

#### MCI

**Fig. 4:**
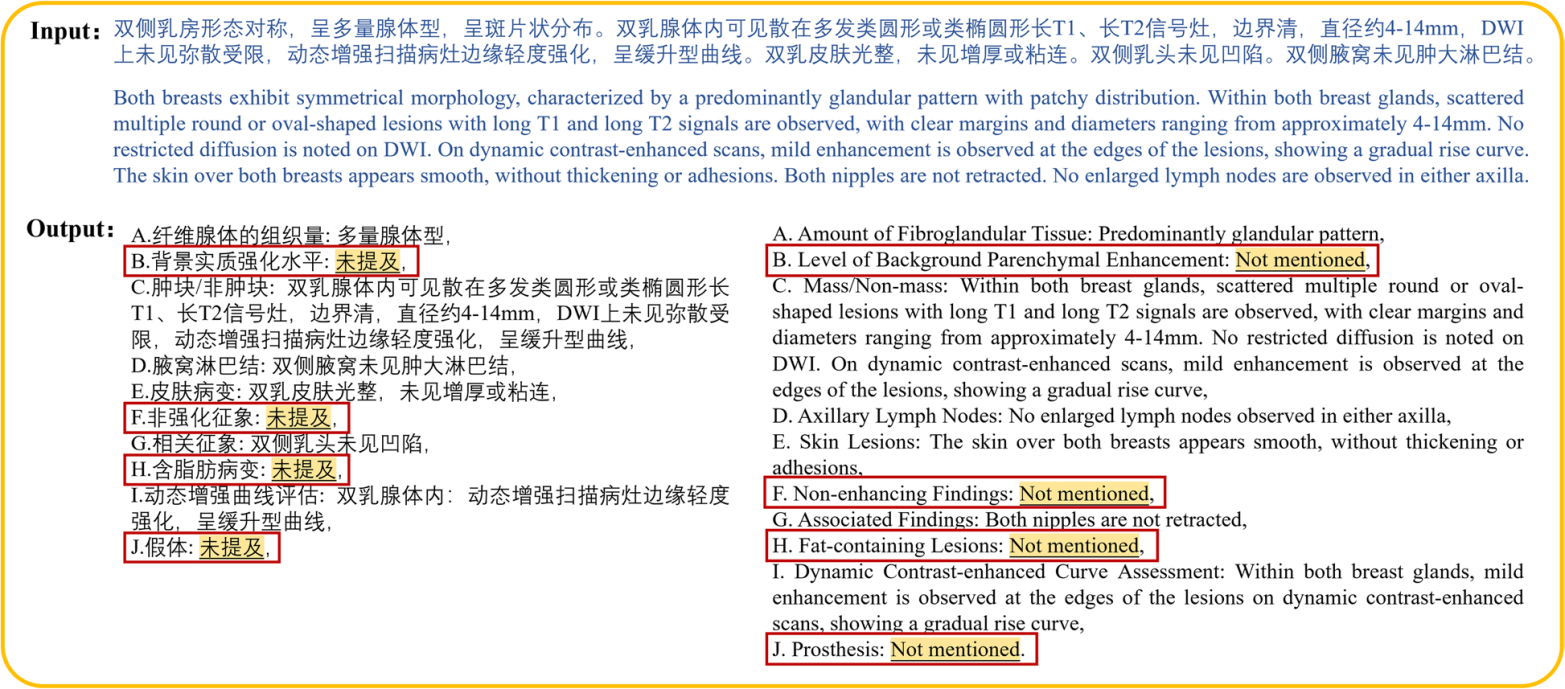
This figure illustrates the process of adding “not mentioned” for missing categories in structured reports. The radiology report content shown is a simplified version created based on real breast MRI reports. The yellow sections indicate the “not mentioned” additions, while the red boxes highlight category information absent from the free-text report. The report examples shown in this figure are English translations of the original Chinese reports

This study employed the Qwen-Chat model to convert free-text reports into structured reports. As shown in Fig. 4, the model extracts information from the free-text report following predefined templates and categorizes it within the corresponding attributes of the structured report. The model incorporates MCI to address features that are absent in the original free-text reports. Following established practices in medical text analysis [30, 36], these missing categories are automatically assigned “not mentioned” as the default value, ensuring consistent handling of undocumented features.

### Integration models

This study proposes a novel fusion strategy based on a transformer model engineered to embed and integrate features from both structured and free-text reports. This approach ensures comprehensive information capture during training. The framework implements a two-stage process: first, both report types undergo embedding encoding and then encoded by the transformer model for feature extraction. Subsequently, the extracted features undergo concatenation and pooling, followed by transformation through a fully connected layer and a softmax function, ultimately producing a prediction corresponding to the sample category.

A transformer model contains a sequence of layers, each containing a multi-head attention mechanism and a feed-forward neural network (FFN) [37] with residual connections and layer normalization. In the multihead attention mechanism, the attention function maps a query and a set of key-value pairs to an output. The input to the attention function consists of query *Q*, key *K*, and value *V*, and is computed as follows:

3 $$\begin{aligned} Attention\left( Q,K,V\right) =softmax\left( \frac{{QK}^{\top }}{\sqrt{{d}_{k}}}\right) V \end{aligned}$$

Here, *Q*, *K*, and *V* represent the query, key, and value, respectively, and ${d}_{k}$ represents the key dimensions. The softmax function calculates the weighted sum of the values using the weights determined by the compatibility function between the query and its corresponding key. The multi-head attention mechanism projects the query, key, and value into multiple subspaces using learned linear projections as follows:

4 $$\begin{aligned} MultiHead\left( {X}^{j}\right) =Concat\left( {head}_{1}, \dots ,{head}_{h}\right) {W}^{O} \end{aligned}$$

Here,

5 $$\begin{aligned} \text {head}_{i}\left( Q,K,V \right) = \text {Attention}\left( X^{j} W_{i}^{Q}, X^{j} W_{i}^{K}, X^{j} W_{i}^{V} \right) \end{aligned}$$

${X}^{j}\in \mathbb {R}^{n*d}$ represents the input representation of sequence j, and ${W}_{i}^{Q}$, ${W}_{i}^{K}$, ${W}_{i}^{V}$, ${W}_{i}^{O}$ are the projection parameter matrices with dimensions $\mathbb {R}^{d*{d}_{k}}$, $\mathbb {R}^{d*{d}_{k}}$, $\mathbb {R}^{d*{d}_{v}}$ and $\mathbb {R}^{h*{d}_{v}*d}$, respectively. In addition to the multihead attention layer, each layer of the model includes an FFN, defined as follows:

6 $$\begin{aligned} FFN\left( {X}^{j}\right) =\max(0, X^jW_1+b_1)W_2+b_2 \end{aligned}$$

where ${W}_{1}$ and ${W}_{2}$ are linear transformation matrices, and b_1_ and b_2_ are the corresponding bias vectors.

## Results

### Datasets

This retrospective study analyzed 11,884 breast MRI reports, which were used as the internal dataset, in Chinese from the Sun Yat-sen Memorial Hospital (SYSMHReports). Additionally, 5043 Chinese reports from the Shantou Central Hospital (SCHReports) were included as the external test dataset. The dataset included MRI reports from multiple anatomical regions, including the brain, breast, thorax, lungs, heart, liver, gallbladder, abdominal cavity, mediastinum, lumbar spine, sacrum, and bladder. For this study, only the reports pertaining to breast and metastatic lesions were considered. Each report comprised two sections: a detailed radiological description and summary of the main findings. This study focused on the detailed radiological description. Expert radiologists with more than five years of clinical experience were invited to annotate the data. Reports were classified into two categories: “Suggestion for Follow-up”, which included lesions classified as BI-RADS 1–3 (benign lesions not typically requiring biopsy), and “Suggestion for Biopsy”, which included lesions classified as BI-RADS 4A-6 (malignant-leaning lesions typically recommended for biopsy). Details of the dataset are listed in Table 1. The internal dataset was randomly split into a 70% training set, 20% testing set, and 10% validation set.

**Table 1:** Details of the datasets

Class	Training set	Validation set	Testing set	External test set	Label
Total	8320	1188	2376	5043	-
Suggestion for follow-up	2119	302	604	1408	0
Suggestion for biopsy	6201	886	1772	3635	1

After referencing the fifth edition of the MRI imaging lexicon [31], radiologists structured the reports into ten categories: amount of fibroglandular tissue, level of background parenchymal enhancement, mass/non-mass, axillary lymph nodes, skin lesions, non-enhancing findings, associated findings, fat-containing lesions, dynamic contrast-enhanced curve assessment, and prosthesis. The details of each category are presented in Table 8 in the Appendix. Approval was obtained from the local Medical Ethics Committee to ensure ethical compliance. The requirement for informed consent was waived due to the use of de-identified data in this study.

### Network training and implementation details

The Qwen-14B-Chat model was initially used to automatically extract information from free-text reports using knowledge-driven prompts, thereby generating structured breast MRI reports. These outputs underwent comprehensive preprocessing, including denoising and review by physicians. The denoising phase employs automated regular expression methods to remove irrelevant symbols and characters followed by physician reviews and corrections. The analysis results identified two main challenges: (1) Insufficient information extraction, which was most prominent in categories such as “associated findings” and “dynamic contrast-enhanced curve evaluation.” This challenge stems primarily from the diverse and heterogeneous content types within these categories, which hinder the accurate extraction of information. (2) Inaccurate information extraction, particularly evident in the “amount of fibroglandular tissue” category. This issue arises from the discrepancy between the clinical descriptions used in real reports and the standardized terminology incorporated into knowledge-driven prompts. To address these challenges, 10,000 screened and organized structured reports were used as a dataset to fine-tune the Qwen-7B-Chat model using the LoRA method. The selection of Qwen-7B-Chat model over Qwen-14B-Chat balanced resource efficiency with performance requirements. This fine-tuned model subsequently processes a second round of inference, targeting previously underperforming data.

This study utilized the Hugging Face Transformer library and PyTorch framework [38, 39] for experimentation. A transformer-based model pre-trained by Google on a large-scale Chinese corpus was utilized for text embedding and fine-tuning to extract textual features from breast MRI reports. The model’s hidden layer had a dimension of *H *= 768, with *A *= 12 attention heads and *L *= 12 transformer layers.

For LoRA fine-tuning of the Qwen-7B-Chat model and all classification experiments, the hardware used consisted of an NVIDIA GPU 3090 (24GB) and an Intel(R) Xeon(R) Gold 6133 CPU @ 2.50GHz. Fine-tuning was conducted with initial learning rates of $3\times 10^{-4}$ and $1\times 10^{-6}$ over 5 and 10 epochs, respectively. Prompt inference using the Qwen-Chat model was performed on a system featuring an NVIDIA GPU A40 (48GB) and a 15-vCPU AMD EPYC 7543 32-Core Processor.

For structuring breast MRI reports, the research strategy proposed by Jeblick et al. [40] was adopted, in which radiologists created 50 virtual breast MRI reports and corresponding structured reference standards. This testing set was used to evaluate the performance of the fine-tuned Qwen-7B-Chat (LoRA) model against other LLMs, including GPT-3.5 [41], GPT-4o [42], and unfine-tuned Qwen-7B-Chat, with virtual reports employed to ensure data privacy. Traditional metrics primarily assess surface-form similarity, which limits their ability to accurately capture the quality of the generated text, particularly in terms of lexical semantics and component diversity. Therefore, this study employed the BERTScore metric [43], which aligns more closely with human judgment, to evaluate the model’s performance in extracting information across the ten categories. BERTScore is computed as follows: for a reference sequence $x= \left\langle {x}_{1},...,{x}_{k}\right\rangle$ and a generated sequence $\hat{x}= \left\langle {\hat{x}}_{1},...,{\hat{x}}_{l}\right\rangle$, the BERT model encodes both sequences to obtain their hidden-layer representations. In this study, a BERT-based Chinese model was used. The F1 score was then calculated as the harmonic mean of precision and recall. For a reference ${x}$ and candidate $\hat{x}$, the recall, precision, and F1 scores are as follows:

7 $$\begin{aligned} {P}_{BERT}= \frac{1}{\left| \hat{x}\right| }\sum \limits _{\hat{{x}_{j}}\in \hat{x}} \max _{{x}_{i}\in x}{{x}_{i}^{\top }{\hat{x}}_{j}} \end{aligned}$$

8 $$\begin{aligned} {R}_{BERT}= \frac{1}{\left| x\right| }\sum \limits _{{x}_{i}\in x} \max _{\hat{{x}_{j}}\in \hat{x}}{{x}_{i}^{\top }{\hat{x}}_{j}} \end{aligned}$$

9 $$\begin{aligned} {F}_{BERT}= 2\frac{{P}_{BERT}\cdot {R}_{BERT}}{{P}_{BERT}+{R}_{BERT}} \end{aligned}$$

To evaluate the effectiveness of the method, ablation and comparative experiments were conducted using different classification models. Several text classification models were tested through comparative experiments to verify the superiority of the proposed method. Representative models from traditional deep-learning methods, including TextCNN [44], TextRCNN [45], and DPCNN [46], were selected. For the transformer models pre-trained on large corpora, MacBERT [47], BERT-wwm [48], BERT-wwm-ext [48], and RoBERTa-wwm-ext [48], were chosen. Additionally, the performance of the Qwen-14B-Chat model in few-shot settings (K = 9) [49], was assessed. The evaluation metrics included precision, recall, F1 score, and area under the curve (AUC).

### Experimental results

#### Result of breast MRI report structuring

Table 2 presents the performance evaluation of the structured reports for extracting information from original reports. Among baseline models, GPT-4o achieved superior performance with the highest ${F}_{BERT}$ of 0.8963. Notably, the LoRA-fine-tuned Qwen-7B-Chat model demonstrated enhanced performance, achieving an ${F}_{BERT}$ of 0.9298, representing a 3.35% improvement. Table 3 details ${F}_{BERT}$ across ten categories in the structured breast MRI reports. The fine-tuned Qwen-7B-Chat model exhibited substantial improvements in multiple categories. However, for certain categories, such as “level of background parenchymal enhancement”, “dynamic contrast-enhanced curve assessment”, and “fat-containing lesions”, the model underperformed compared to the GPT-4o.

**Table 2:** Evaluation results of structured breast MRI for various models

Model	$\varvec{P}_{\varvec{BERT}}$	$\varvec{R}_{\varvec{BERT}}$	$\varvec{F}_{\varvec{BERT}}$
Qwen-7B-Chat	0.8033	0.8127	0.8080
Qwen-14B-Chat	0.8395	0.8356	0.8376
GPT-3.5	0.8690	0.8914	0.8801
GPT-4o	0.8868	0.9059	0.8963
Qwen-7B-Chat (Fine-tuned)	**0.9381**	**0.9217**	**0.9298**

The best results are highlighted in bold

**Table 3:** ${F}_{BERT}$ for 10 categories obtained via different methods in structured breast MRI reports

Category	Qwen-7B-Chat	Qwen-14B-Chat	GPT-3.5	GPT-4o	Qwen-7B-Chat (Fine-tuned)
Amount of fibroglandular tissue	0.7145	0.6166	0.7901	0.8259	**0.9490**
Level of background parenchymal enhancement	0.8711	**0.9976**	0.9700	0.9801	0.9623
Mass/non-mass	0.7849	0.8423	0.8877	0.9133	**0.9443**
Axillary lymph nodes	0.8883	0.9530	0.9734	0.9728	**0.9787**
Skin lesions	0.6720	0.7608	0.7923	0.7866	**0.9149**
Non-enhancing findings	0.9069	0.9357	0.9131	0.9391	**0.9437**
Associated findings	0.7177	0.6669	0.7461	0.7770	**0.8900**
Fat-containing lesions	0.9157	0.9707	0.9821	**0.9831**	0.9749
Dynamic contrast-enhanced curve assessment	0.6821	0.7863	0.8039	**0.8455**	0.7523
Prosthesis	0.9065	0.9219	0.9192	0.9205	**0.9731**

The best results are highlighted in bold

**Fig. 5:**
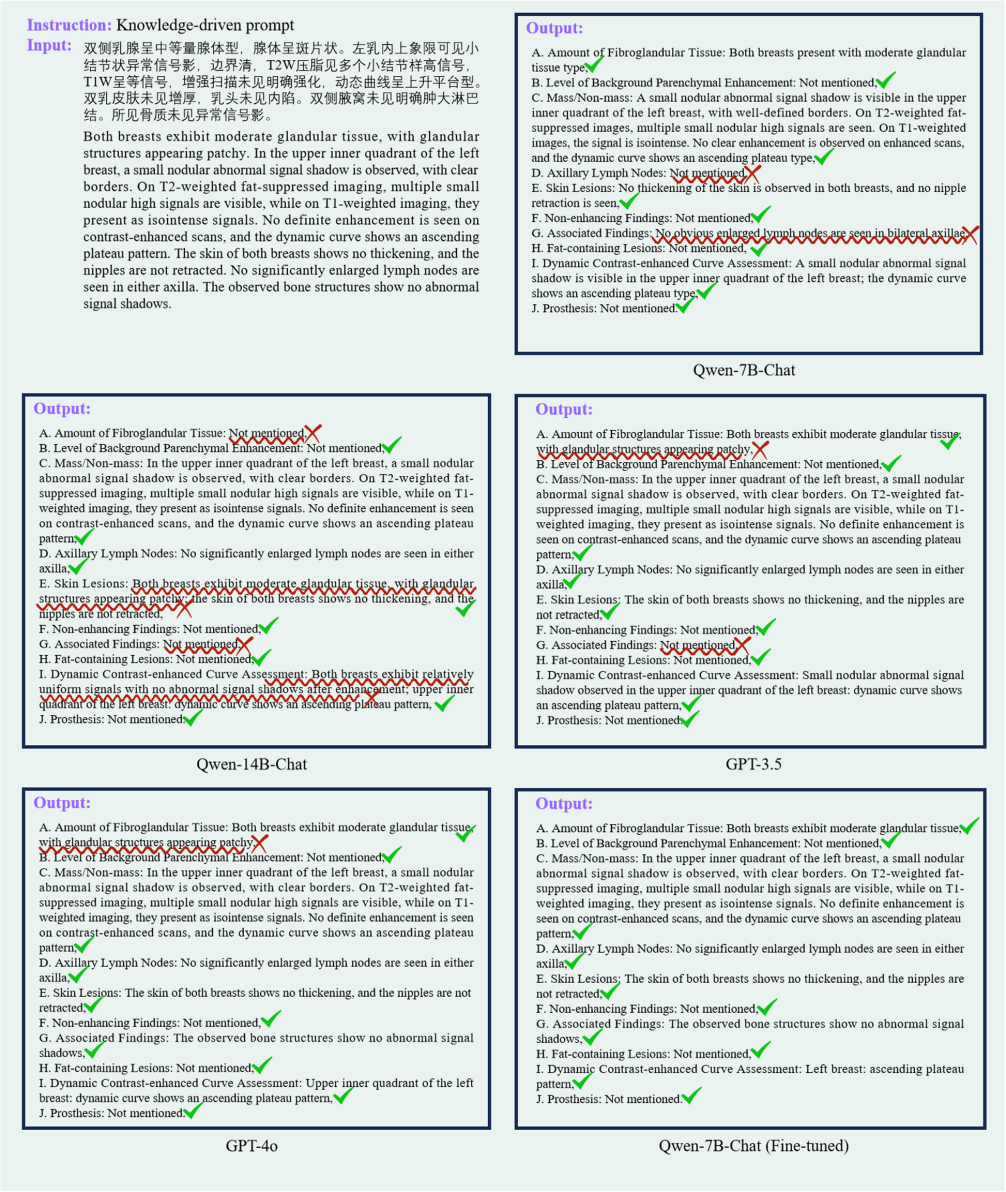
Comparison of different model outputs. Red wavy lines in the figure indicate the occurrence of information extraction errors. The “red cross mark” denotes an error in information extraction, while the “green check mark” denotes correct information extraction. Each structured output shown is translated from the original Chinese reports

Figure 5 illustrates the inference results of each model for a virtual report, with the results denoised and translated into English. Red crosses and wavy red lines highlight errors in the extraction, whereas green checks indicate accurate semantic information extraction. Compared with the online GPT models, the results from direct inference using Qwen-7B-Chat and Qwen-14B-Chat showed more errors. However, the fine-tuned Qwen-7B-Chat model significantly improved the accuracy of information extraction.

#### Result of breast MRI report classification

The proposed method was evaluated using both an internal test set (SYSMHReports) and an external test set (SCHReports). Table 4 lists the four evaluation metrics for the various comparison methods. The proposed method achieved the highest precision, recall, F1 score, and AUC values for both datasets. Among the compared methods, transformer-based models exhibited the second-best overall performance. Specifically, the BERT-wwm model demonstrated the second-best recall, F1 score, and AUC on the SYSMHReports dataset and the second-best precision, recall, and F1 score on the SCHReports dataset. The BERT-wwm-ext model achieved the second-best precision on the SYSMHReports dataset. As a representative of traditional deep learning methods, TextCNN performed well on SYSMHReports, whereas TextRCNN excelled on SCHReports. The TextCNN model achieved the second best AUC for the SCHReports dataset. In contrast, the few-shot learning performance of Qwen-14B-Chat was approximately 10% lower compared to the other models.

**Table 4:** Classification performance of various models on the test set

Model	SYSMHReport				SCHReport			
	Precision	Recall	F1 score	AUC	Precision	Recall	F1 score	AUC
Traditional deep learning model								
TextRCNN [45]	0.8599	0.8628	0.8537	0.8944	0.8425	0.8435	0.8430	0.9041
TextCNN [44]	0.8721	0.8742	0.8662	0.9085	0.8588	0.8620	0.8593	0.9208
DPCNN [46]	0.8653	0.8683	0.8606	0.9086	0.8529	0.8562	0.8499	0.9088
Transformer model								
MacBERT [47]	0.8563	0.8603	0.8518	0.9007	0.8473	0.8431	0.8448	0.9073
RoBERTa-wwm-ext [48]	0.8626	0.8653	0.8567	0.9177	0.8496	0.8517	0.8504	0.9149
BERT-wwm-ext [48]	0.8744	0.8746	0.8658	0.9320	0.8603	0.8612	0.8607	0.9152
BERT-wwm [48]	0.8733	0.8758	0.8693	0.9324	0.8653	0.8626	0.8637	0.9165
LLM (few-shot learning)								
Qwen-14B-Chat [32]	0.7461	0.7377	0.7419	-	0.7209	0.7061	0.7134	-
Ours	**0.9003**	**0.9024**	**0.9000**	**0.9542**	**0.8759**	**0.8665**	**0.8694**	**0.9295**

The best results are highlighted in bold

#### Ablation study

Several ablation studies were conducted and the corresponding analyses were provided.

#1: Effects of knowledge-driven prompt. The MRI lexicon was removed from the knowledge-driven prompts, and the performance of the Qwen-14B-Chat model was evaluated using the default prompts. Figure 6 presents the performance results for the different categories. The experiments demonstrated that the knowledge-driven prompt significantly improved the information extraction performance for most categories, effectively mitigating the risk of extracting irrelevant information owing to literal interpretations of category names, as illustrated in Fig. 7. However, the performance of the model exhibited a notable degradation in certain categories. Complete examples are provided in Table 9 in the Appendix.

**Fig. 6:**
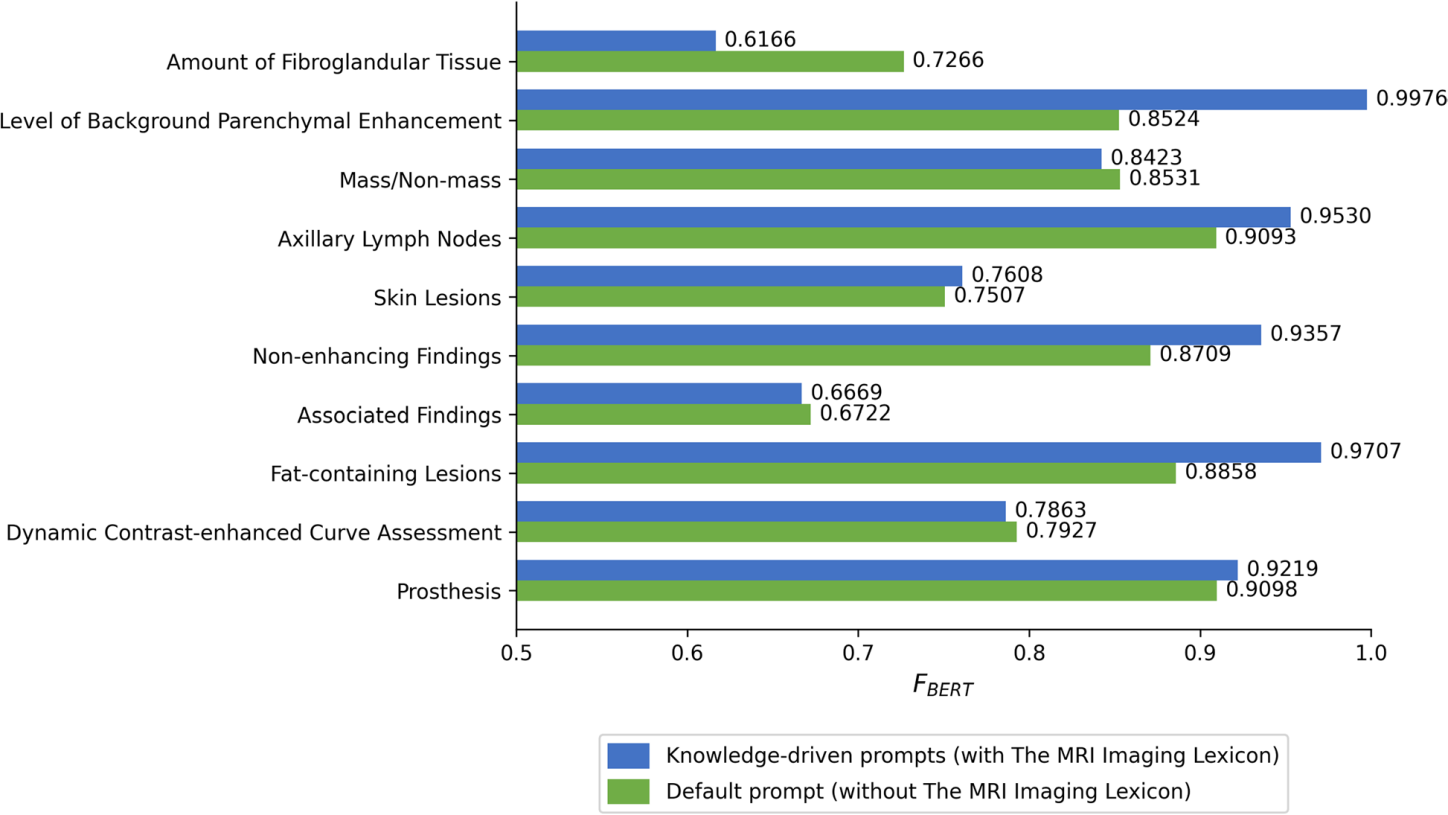
Information extraction performances of Qwen-14B-Chat model with different prompts

**Fig. 7:**
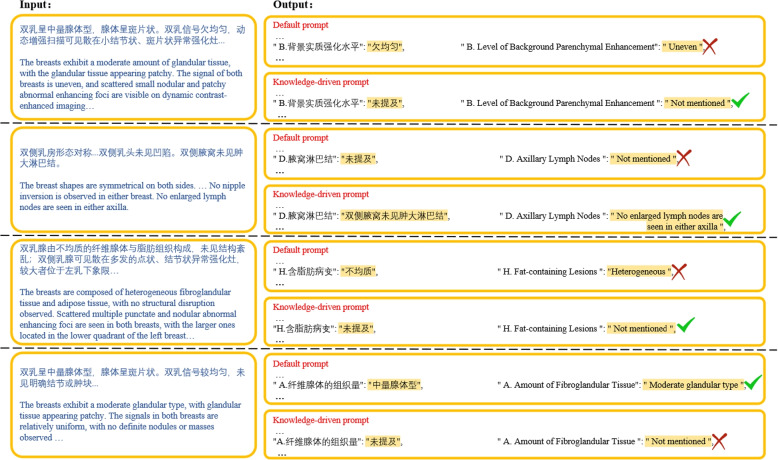
Effect of using knowledge-driven prompts on free-text reports. The “red cross mark” denotes incorrect information extraction, while the “green check mark” denotes correct information extraction. The reports shown are the English translations of the original Chinese reports. Free-text and structured reports are shown in truncated form

#2: Effect of in-context example quantity. The impact of varying the number of in-context examples on the performance of the Qwen-14B-Chat in structured information extraction from breast MRI reports was extracted. As shown in Table 5, the model’s performance consistently improved as we increased the number of examples from 0 to 5, with the accuracy increasing from 0.7178 to 0.8376. However, a slight decline in performance was observed when the number of examples was further increased to 7.

**Table 5:** Evaluation results of Qwen-14B-Chat on structured breast MRI reports with different numbers of in-context examples

Number of example	$\varvec{P}_{\varvec{BERT}}$	$\varvec{R}_{\varvec{BERT}}$	$\varvec{F}_{\varvec{BERT}}$
0	0.7352	0.7012	0.7178
1	0.8065	0.7993	0.8029
3	0.8277	0.8228	0.8253
5	**0.8395**	**0.8356**	**0.8376**
7	0.8234	0.8110	0.8172

The best performance is highlighted in bold

#3: Effects of MCI. The MCI was removed from the structured reports, and the model was trained using structured reports to assess its performance on the SYSMHReports dataset. The first section of Table 6 summarizes the performance of the model in terms of precision, recall, F1 score, and AUC. The results indicate that when MCI is included, the model’s F1 score improves to 0.8865 (+2.46%) and AUC increases to 0.9405 (+2.83%). Figure 8a shows a visualization of the model’s weight assignment to a structured report, where the “not mentioned” areas are highlighted in darker colors, indicating a higher weight assignment.

**Table 6:** Performance analysis of report formats for BI-RADS classification on the SYSMHReports dataset

Index	Precision	Recall	F1 score	AUC
Structured report				
*Without MCI*	0.8687	0.8704	0.8619	0.9122
* With MCI*	**0.8862**	**0.8889**	**0.8865**	**0.9405**
Free-text report				
* Without PH*	0.8687	0.8708	0.8628	0.9196
* With PH*	0.8710	0.8729	0.8652	0.9311

The best results are highlighted in bold

*PH* Personal history

**Fig. 8:**
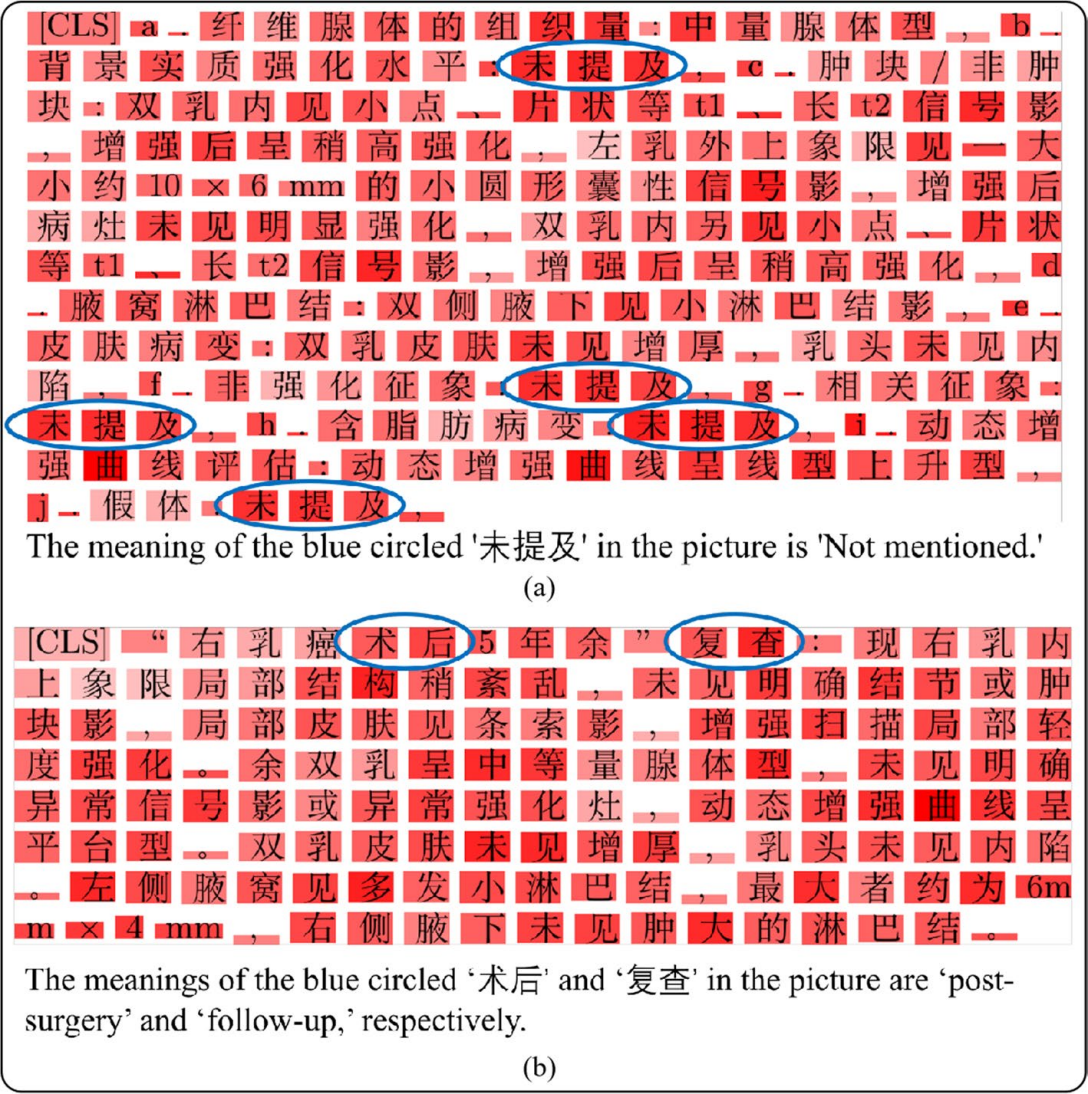
Visualization of attention weights assigned to a sample structured and free-text report by the model. Words with higher weights are shown in darker red, indicating greater importance to the model

#4: Effects of PH. During the conversion of free-text reports to structured reports, a subtle yet important phenomenon was observed. Owing to the absence of the “personal history” category in the template (as shown in Fig. 9), LLMs were employed to automatically extract the PH. After removing PH from the free-text reports, the model was trained using free-text reports, and its performance was evaluated on the SYSMHReports dataset. The second section of Table 6 presents the performance of the model in terms of precision, recall, F1 score, and AUC. The results indicate that including PH improves the AUC to 0.9311 (+1.15%). Figure 8b visualizes the model’s weight distribution for a free-text report with sections related to PH (e.g., “post-surgery” and “follow-up”) highlighted in darker colors, signifying higher weight. From a clinical perspective, PH plays a vital role in breast cancer MRI screening [50–52].

**Fig. 9:**
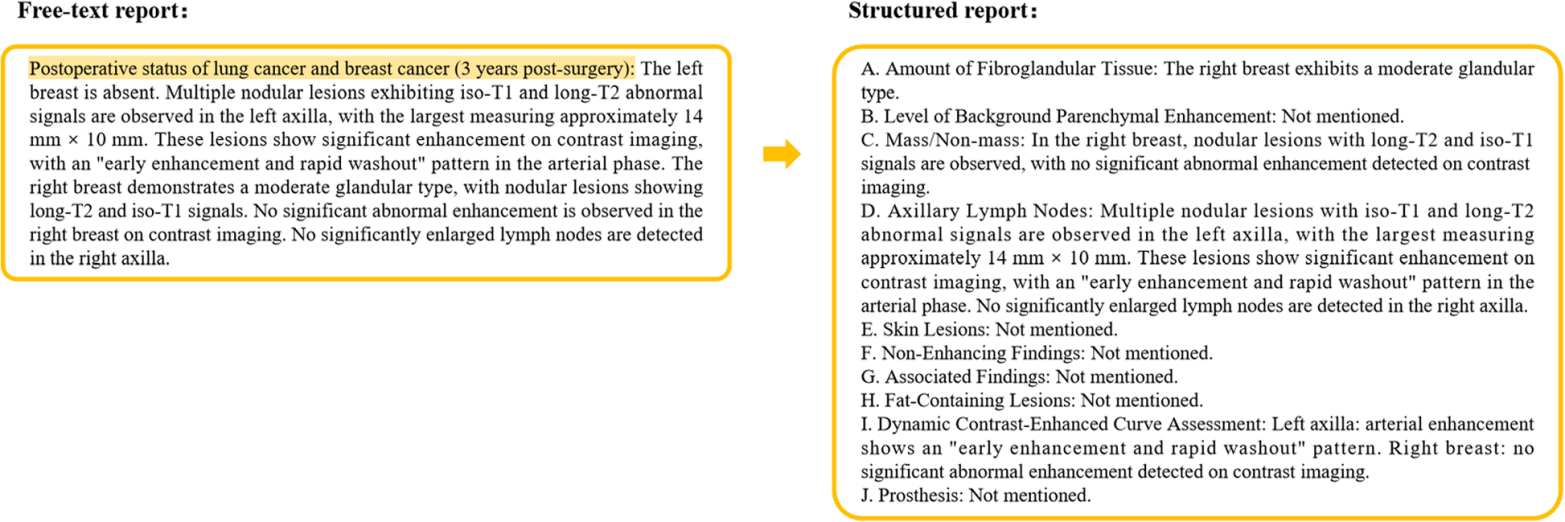
PH in a free-text report. When a free-text report is converted to a structured report, the PH is lost (the PH highlighted in yellow). The reports shown are the English translations of the original Chinese reports

#5: Effects of fusion strategy. Table 7 presents the model performance for various data fusion strategies. The experimental results show that the fusion strategies significantly improved the model performance compared to training with structured reports or free-text reports individually. Notably, the proposed concatenation fusion strategy achieved the best performance in terms of precision, recall, F1 score, and AUC.

**Table 7:** Comparison of model performance with and without fusion, as well as under alternative fusion strategies

Fusion strategy	Input		Index			
	Free-text report	Structured report	Precision	Recall	F1 score	AUC
Without fusion	✓		0.8710	0.8729	0.8652	0.9311
Without fusion		✓	0.8862	0.8889	0.8865	0.9405
Cross-attention fusion	✓	✓	0.8874	0.8902	0.8870	0.9453
Average-pooling fusion	✓	✓	0.8964	0.8986	0.8956	0.9502
Addition fusion	✓	✓	0.8947	0.8969	0.8938	0.9511
Max-pooling fusion	✓	✓	0.8961	0.8981	0.8948	0.9513
**Concatenation fusion (ours)**	✓	✓	**0.9003**	**0.9024**	**0.9000**	**0.9542**

The best results are highlighted in bold

## Discussion

This study proposed a novel BI-RADS classification method for breast MRI reports that thoroughly explored the information contained within the reports. Comprehensive experimental results demonstrated that the proposed approach outperformed the baseline methods in terms of reporting classification performance. Ablation studies highlighted the critical significance of the MCI.

During report structuring, the introduced knowledge-driven prompts effectively enhanced the extraction of category information across most classes. However, certain categories posed challenges, as the model struggled to fully leverage prior knowledge. This limitation was due to the disparity between intuitive clinical descriptions and strict medical terminology, leading to mismatches between real-world reports and predefined terms. Model fine-tuning successfully addressed these limitations. The robust performance of knowledge-driven prompts across most categories provides a solid foundation for further optimization of the prior knowledge system and continued enhancement of model learning performance.

Although the proposed fusion strategy demonstrates promising performance, it required accommodating a degree of information redundancy when merging structured reports with free-text reports to ensure the capture of comprehensive clinical information. Future work will aim to refine this approach by developing more efficient fusion mechanisms that minimize redundancy while maintaining information completeness, thereby enhancing model efficiency and performance.

The optimization of example quantities in prompts was investigated. The results show that the performance significantly improved as the number of examples increased from 0 to 5, demonstrating substantial gains in accuracy. However, when the number of examples was further increased to 7, a slight decline in performance was observed. This finding reveals that simply increasing the number of examples is not an optimal strategy. Experimental results indicate that, under the constraints of limited context windows, an excessive number of examples can dilute the model’s attention and affect its focus on tasks. In particular, for domain-specific tasks, it was found that a moderate set of examples was sufficient to establish the necessary task patterns and achieve optimal performance.

Despite the limited sample size of the real-world dataset, the model exhibited exceptional performance, highlighting its significant potential for large-scale training with datasets from additional centers in the future. Although this study focused on single-modal text data, existing research has demonstrated that multimodal learning can integrate information from different sources to enhance model understanding [53–55]. Future research could explore the combination of textual data with medical images to develop more efficient multimodal methods for improving medical classification decisions.

In recent years, artificial intelligence has demonstrated extensive applicability in clinical decision support, disease diagnosis, and health monitoring [56]. As a cutting-edge artificial intelligence technology, LLMs offer promising opportunities to address challenges in the medical field. Although LLMs have provided significant advances and convenience, the substantial memory and computational resources required for fine-tuning remain major obstacles to their widespread application. Additionally, the effectiveness of LLM fine-tuning depends heavily on data quality, which can significantly impact model performance and robustness. Similar to the image and video quality assessments [57–60], text data quality evaluation is crucial. While current data screening and evaluation still rely on manual operations, future work will focus on developing automated quality assessment methods to optimize the text data screening process, thereby better addressing the clinical needs in practice.

## Conclusions

This study presented a BI-RADS classification method leveraging LLMs and transformer models to thoroughly explore information from breast MRI reports. This method incorporated the MCI by converting free-text reports into structured reports, thereby effectively enriching the learning content of the model. To ensure data privacy and enhance the adaptability of LLMs in specialized domains, LLMs were deployed locally, and a knowledge-driven prompt was designed. To improve the capability of the model in structuring breast MRI reports, targeted fine-tuning was conducted. Furthermore, to ensure the comprehensiveness and diversity of the training data, a fusion strategy was proposed to synergistically utilize information from both structured and free-text reports. Compared with other baseline methods, the proposed approach achieved significant advantages in reporting classification tasks. The ablation studies verified the influence of each component. Additionally, the proposed method was evaluated using datasets from two independent centers, and the experimental results demonstrated its robustness and reliability.

## Supplementary information

Supplementary Material 1.

## Data Availability

The clinical data used in this research, SYSMHReports, were provided by Sun Yat-sen Memorial Hospital, and SCHReports were provided by Shantou Central Hospital. Clinical data are not publicly available as they contain private patient health information. To ensure ethical compliance, approval was obtained from the local medical ethics committee. The requirement for informed consent was waived due to the use of de-identified data in this study.

## Abbreviations

MRI: Magnetic resonance imaging
MCI: Missing category information
NLP: Natural language processing
SVM: Support vector machine
KNN: K-nearest neighbor
NB: Naive Bayes
CNN: Convolutional neural network
RNN: Recurrent neural network
BERT: Bidirectional encoder representations from transformers
LLM: Large language model
GLM: Generative language model
LoRA: Low-rank adaptation
FFN: Feed-forward neural network
AUC: Area under the curve
PH: Personal history
SYSMHReports: Sun Yat-sen Memorial Hospital Breast MRI Reports
SCHReports: Shantou Central Hospital Breast MRI Reports
