# Supplementary material for: Bootstrapping BI-RADS classification using large language models and transformers in breast magnetic resonance imaging reports
Source: Vis Comput Ind Biomed Art. 2025 Apr 3;8:8. doi: 10.1186/s42492-025-00189-8 (PMC11968601; doi:10.1186/s42492-025-00189-8)
Supplement: Supplementary file 1 — Supplementary Material 1. [file 42492_2025_189_MOESM1_ESM.docx]

**Bootstrapping BI-RADS Classification Using LLMs and Transformers in Breast MRI Reports**

Supplementary Material

**Table 8** Integration of the 5th Edition MRI Imaging Lexicon in Structured Report Categories.

| Categories | Description Based on the 5th MRI Imaging Lexicon |
| --- | --- |
| A. Amount of Fibroglandular Tissue | Class A (almost entirely fatty tissue) / Class B (scattered fibroglandular tissue) / Class C (unevenly distributed fibroglandular tissue) / Class D (dense fibroglandular tissue) |
| B. Level of Background Parenchymal Enhancement | Level: Minimal/Mild/Moderate/Marked  Symmetry: Symmetric/Asymmetric |
| C. Mass/Non-mass | Small and isolated enhancement foci <5mm, punctate; Mass/Mass-like enhancement; Focal: Limited to one quadrant of the breast and confined to a single ductal system; Linear: Linear enhancement corresponding to a single ductal system; Segmental: Triangular enhancement with the apex pointing toward the nipple; Regional: Involving at least one quadrant, encompassing a broader region than a single ductal system, potentially map-like with a lack of convex contours  Multiple Regions: At least two large areas separated by normal glandular/fatty tissue, diffusely distributed, extensive, and uniform |
| D. Axillary Lymph Nodes | Axillary lymphadenopathy |
| E. Skin Lesions | Skin lesion/Keloid/ Sebaceous cyst/Dermatitis/Skin retraction/Skin thickening/Skin invasion (direct invasion or inflammatory carcinoma) |
| F. Non-enhancing Findings | T1W hyperintensity/ Cyst/ Postoperative hematoma or seroma/ Post-treatment skin thickening and trabecular thickening/ Non-enhancing mass/ Structural distortion/ Signal void caused by implants, markers, or other artifacts |
| G. Associated Findings | Nipple retraction/ Nipple invasion/ Pectoralis muscle invasion/ Chest wall invasion/ Structural distortion |
| H. Fat-containing Lesions | Fat necrosis/ Hamartoma/ Postoperative fat-containing seroma or seroma |
| I. Dynamic Contrast-enhanced Curve Assessment | Early phase: Slow/Intermediate/Rapid  Delayed phase: Persistent/Plateau/Washout |
| J. Prosthesis | Implant material and type: Saline/Silicone/Other materials/Type; Integrity: Intact/Ruptured; Implant location: Retroglandular/Retromuscular; Abnormal implant contour: Focal bulging; Intracapsular silicone implant abnormality: Radial folds/Subcapsular lines/Keyhole sign/Linguine sign; Extracapsular silicone: Intraparenchymal/Intralymphatic; Water droplet sign: Not observed/Observed; Peri-implant fluid: Not observed/Observed |

**Table 9** Examples of Structured Reports Using Default Prompt and Knowledge-driven Prompt. Red marks indicate information extraction errors.

| Free-text Report | Structured Reports  (Default Prompt) | Structured Reports (Knowledge-driven Prompt) |
| --- | --- | --- |
| The bilateral breasts consist of heterogeneous fibroglandular and fatty tissue, with no structural distortion. Multiple scattered punctate and nodular areas of abnormal enhancement are seen in both breasts, with the largest one located in the lower quadrant of the left breast, measuring approximately 8mm in diameter, with clear borders. The adjacent glandular tissue shows no convergence or distortion. Dynamic enhancement shows progressive enhancement pattern, with no significant early enhancement, and the time-signal intensity curve shows a persistent pattern. The retromammary space is clear bilaterally, and the pectoralis major muscles show no abnormal signal. Bilateral Cooper's ligaments show no thickening, the skin is not thickened, and there is no nipple retraction bilaterally. No enlarged lymph nodes are seen in either axilla. | A. Amount of Fibroglandular Tissue: Heterogeneous,  B. Level of Background Parenchymal Enhancement: Not mentioned,  C. Mass/Non-mass Enhancement: Multiple scattered punctate and nodular areas of abnormal enhancement seen bilaterally, with the largest one in the lower quadrant of left breast, measuring approximately 8mm in diameter, with clear borders. Adjacent glandular tissue shows no convergence or distortion. Dynamic enhancement shows progressive pattern, with no significant early enhancement, and time-signal intensity curve shows persistent pattern,  D. Axillary Lymph Nodes: No enlarged lymph nodes bilaterally,  E. Skin Lesions: No skin thickening,  F. Non-enhancing Findings: Not mentioned,  G. Associated Findings: Clear retromammary space, no abnormal signal in bilateral pectoralis major muscles H. Fat-containing Lesions: Heterogeneous,  I. Dynamic Contrast-enhanced Curve Assessment: Progressive enhancement pattern, no significant early enhancement,  J. Prosthesis: Not mentioned; | A. Amount of Fibroglandular Tissue: Not mentioned,  B. Level of Background Parenchymal Enhancement: Not mentioned,  C. Mass/Non-mass Enhancement: Multiple scattered punctate and nodular areas of abnormal enhancement seen bilaterally, with the largest one in the lower quadrant of left breast, measuring approximately 8mm in diameter, with clear borders. Adjacent glandular tissue shows no convergence or distortion. Dynamic enhancement shows progressive pattern,  D. Axillary Lymph Nodes: No enlarged lymph nodes bilaterally,  E. Skin Lesions: No skin thickening bilaterally,  F. Non-enhancing Findings: Not mentioned,  G. Associated Findings: Clear bilateral retromammary space, no thickening of bilateral Cooper's ligaments,  H. Fat-containing Lesions: Not mentioned,  I. Dynamic Contrast-enhanced Curve Assessment: Progressive enhancement pattern,  J. Prosthesis: Not mentioned; |
| The bilateral breasts are symmetrical in shape, showing abundant glandular pattern with patchy distribution. Multiple scattered round or oval-shaped lesions with high T1 and T2 signal intensity are visible within the breast tissue, with clear borders, measuring approximately 4-14mm in diameter. No diffusion restriction is seen on DWI, and dynamic enhancement scanning shows mild enhancement at the lesion margins with persistent-type curve. The bilateral breast skin is smooth without thickening or adhesion. No nipple retraction is noted bilaterally. No enlarged lymph nodes are seen in either axilla. | A. Amount of Fibroglandular Tissue: Abundant glandular pattern,  B. Level of Background Parenchymal Enhancement: Not mentioned,  C. Mass/Non-mass Enhancement: Multiple scattered round or oval-shaped lesions with high T1 and T2 signal intensity, clear borders, measuring approximately 4-14mm in diameter. No diffusion restriction on DWI, mild enhancement at lesion margins on dynamic enhancement scanning with persistent-type curve,  D. Axillary Lymph Nodes: Not mentioned,  E. Skin Lesions: Smooth bilateral breast skin, no thickening or adhesion,  F. Non-enhancing Findings: Not mentioned,  G. Associated Findings: Not mentioned,  H. Fat-containing Lesions: Not mentioned,  I. Dynamic Contrast-enhanced Curve Assessment: Multiple scattered round or oval-shaped lesions with high T1 and T2 signal intensity: mild enhancement at lesion margins on dynamic scanning with persistent-type curve,  J. Prosthesis: Not mentioned; | A. Amount of Fibroglandular Tissue: Not mentioned,  B. Level of Background Parenchymal Enhancement: Not mentioned,  C. Mass/Non-mass Enhancement: Multiple scattered round or oval-shaped lesions with high T1 and T2 signal intensity seen in bilateral breasts, clear borders, measuring approximately 4-14mm in diameter. No diffusion restriction on DWI, mild enhancement at lesion margins on dynamic enhancement scanning with persistent-type curve,  D. Axillary Lymph Nodes: No enlarged lymph nodes bilaterally,  E. Skin Lesions: No nipple retraction bilaterally,  F. Non-enhancing Findings: Not mentioned,  G. Associated Findings: No nipple retraction bilaterally,  H. Fat-containing Lesions: Not mentioned,  I. Dynamic Contrast-enhanced Curve Assessment: Multiple scattered round or oval-shaped lesions with high T1 and T2 signal intensity: mild enhancement at lesion margins on dynamic scanning with persistent-type curve,  J. Prosthesis: Not mentioned; |
| The breasts show moderate glandular pattern with patchy distribution. The signal is heterogeneous bilaterally. Dynamic enhancement scanning reveals scattered small nodular and patchy areas of abnormal enhancement, showing a "persistent" enhancement pattern. No skin thickening is seen, and there is no nipple retraction. No enlarged lymph nodes are noted in either axilla. | A. Amount of Fibroglandular Tissue: Moderate glandular pattern bilaterally with patchy distribution,  B. Level of Background Parenchymal Enhancement: Heterogeneous,  C. Mass/Non-mass Enhancement: Heterogeneous signal bilaterally, scattered small nodular and patchy areas of abnormal enhancement on dynamic enhancement scanning, showing persistent enhancement pattern,  D. Axillary Lymph Nodes: No enlarged lymph nodes bilaterally,  E. Skin Lesions: No skin thickening, no nipple retraction,  F. Non-enhancing Findings: Not mentioned,  G. Associated Findings: Not mentioned,  H. Fat-containing Lesions: Not mentioned,  I. Dynamic Contrast-enhanced Curve Assessment: Heterogeneous signal bilaterally, scattered small nodular and patchy areas of abnormal enhancement on dynamic scanning, showing persistent enhancement pattern,  J. Prosthesis: Not mentioned; | A. Amount of Fibroglandular Tissue: Not mentioned,  B. Level of Background Parenchymal Enhancement: Not mentioned,  C. Mass/Non-mass Enhancement: Patchy pattern bilaterally, scattered small nodular and patchy areas of abnormal enhancement on dynamic enhancement scanning,  D. Axillary Lymph Nodes: No enlarged lymph nodes bilaterally,  E. Skin Lesions: No skin thickening, no nipple retraction,  F. Non-enhancing Findings: Not mentioned,  G. Associated Findings: Not mentioned,  H. Fat-containing Lesions: Not mentioned,  I. Dynamic Contrast-enhanced Curve Assessment: Patchy pattern bilaterally, scattered small nodular and patchy areas of abnormal enhancement on dynamic scanning: showing 'persistent-type' enhancement pattern,  J. Prosthesis: Not mentioned; |
